# Fast Screening of Antibacterial Compounds from Fusaria

**DOI:** 10.3390/toxins8120355

**Published:** 2016-11-29

**Authors:** Teis Esben Sondergaard, Marlene Fredborg, Ann-Maria Oppenhagen Christensen, Sofie K. Damsgaard, Nikoline F. Kramer, Henriette Giese, Jens Laurids Sørensen

**Affiliations:** 1 Department of Chemistry and Bioscience, Aalborg University, Frederik Bajers Vej 7H, DK-9220 Aalborg Ø, Denmark; a-mariac@hotmail.com (A.-M.O.C.); baldurskd@hotmail.com (S.K.D.); nikolinekramer@hotmail.com (N.F.K.); hgiese@bio.aau.dk (H.G.); jls@bio.aau.dk (J.L.S.); 2 Department of Animal Science, Faculty of Science and Technology, Aarhus University, Blichers Allé 20, DK-8830 Tjele, Denmark; marlene.fredborg@anis.au.dk; 3 Department of Chemistry and Bioscience, Aalborg University, Niels Bohrs Vej 8, 6700 Esbjerg, Denmark

**Keywords:** *Fusarium*, mycotoxins, secondary metabolites, bioactivity, polyketides, pigments, antibiotics, bio-guided assays, antibacterial, aurofusarin

## Abstract

Bio-guided screening is an important method to identify bioactive compounds from fungi. In this study we applied a fast digital time-lapse microscopic method for assessment of the antibacterial properties of secondary metabolites from the fungal genus *Fusarium*. Here antibacterial effects could be detected for antibiotic Y, aurofusarin, beauvericin, enniatins and fusaric acid after six hours of cultivation. The system was then used in a bio-guided screen of extracts from 14 different *Fusarium* species, which had been fractionated by HPLC. In this screen, fractions containing the red pigments aurofusarin and bikaverin showed effects against strains of *Lactobacillus* and *Bifidobacterium*. The IC_50_ for aurofusarin against *Lactobacillus acidophilus* was 8 µM, and against *Bifidobacterium breve* it was 64 µM. Aurofusarin only showed an effect on probiotic bacteria, leading to the speculation that only health-promoting bacteria with a positive effect in the gut system are affected.

## 1. Introduction

Filamentous fungi represent a rich source of secondary metabolites used in the battle for survival in natural habitats and as infection facilitators during host pathogenesis. Each fungus has a specific arsenal intended to inhibit growth of competing organisms such as bacteria. Several antibacterial compounds have been isolated from fungi, with penicillin, produced by *Penicillium* and *Aspergillus* species, being the most famous [1]. In the search for novel antibacterial secondary metabolites, several bio-guided approaches can be applied such as fractionating the secondary metabolome by reverse-phase liquid chromatography [2,3] or by explorative solid-phase extraction [4,5]. The fractions are then often assessed for bioactivity through traditional techniques (e.g., spectrophotometry). The recent development in real-time monitoring systems has enabled accurate bacterial susceptibility testing within a few minutes or hours [6].

In the present study we used the oCelloScope real-time microscopy system to detect antibacterial effects of compounds from *Fusarium* species. The *Fusarium* genus comprises a group of pathogenic fungi that are found throughout the world. Some *Fusarium* species are able to colonize a wide range of plant species, whereas others are pathogens of insects and mammals. Members of the genus contain typically more than 30 different secondary metabolite gene clusters, of which several are species-specific [7]. Some secondary metabolites from *Fusarium* have been shown to possess an antibacterial effect, including antibiotic Y, beauvericin, enniatins and fusaric acid [8,9,10,11,12,13,14,15,16,17,18,19,20,21]. Others such as T-2 toxin, diacetoxyscirpenol and deoxynivalenol have not been shown to have an antibacterial effect [22]. There is a huge potential to discover novel secondary metabolites in *Fusarium* as the genetic information shows that only one-fourth of the potentially produced compounds have been identified.

## 2. Results and Discussion

Seventeen available secondary metabolites known to be produced by *Fusarium* were used in an initial experiment to set up the oCelloScope system for screening for antibacterial activity of fungal compounds against *Lactobacillus acidophilus*, *Escherichia coli*, *Staphylococcus aureus* and *Salmonella typhimurium* (Supplementary Figures S1–S4). In this screen, fusaric acid, beauvericin, enniatins, antibiotic Y and aurofusarin exhibited antibacterial activity against one or more of the four bacterial species. To evaluate IC_50_ values for the five secondary metabolites with antibacterial potential, we used the oCelloScope system armed with the SESA algorithm, which is optimized to detect bacterial growth in liquid suspensions when the total bacteria number is low (Table 1).

Strong inhibitions of bacterial growth could be determined within the first hour of the experiment, whereas milder inhibitions were observed after 4–6 h of incubation (Video S1). Visual inspection of the generated movies could also be used to detect precipitation of antibiotic Y at high concentrations (Video S1).

The most potent compound was aurofusarin with an IC_50_ value of 8 µM against *L. acidophilus*. Antibiotic Y, beauvericin, enniatins, and fusaric acid had weak antibiotic effects, in accordance with other studies [8,9,10,11,12,13,14,15,16,17,18,19,20,21]. Comparison of IC_50_ or MIC values between different studies is never straightforward due to differences in methods, solubility and purity of the tested compounds. We did not observe any antibiotic effect of the 12 remaining compounds: cyclosporine, diacetoxyscirpenol, deoxynivalenol, fumonisin B1, fusarin C, fusarielin A, fusarielin H, moniliformin, nivalenol, neosolaniol, T-2 toxin, and zearalenone (Figures S1–S4); this is also in agreement with earlier reports. Cyclosporine has been reported to enhance the ability of *E. coli* to stick to human epithelial cells [23]. Fusarielin H has not been tested before and showed no effect.

**Figure 1:**
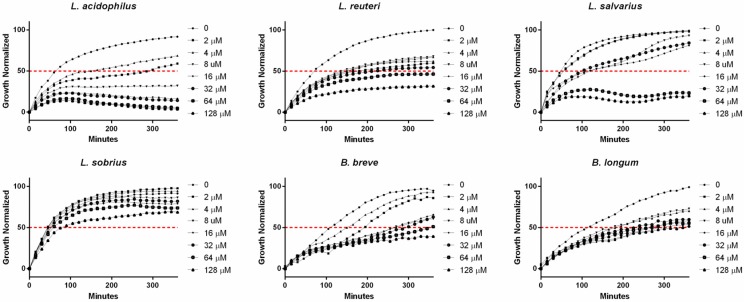
The inhibition effects of aurofusarin on *Lactobacillus* spp. and *Bifidobacterium* spp. Aurofusarin was tested in concentrations between 2 and 128 µM. The IC_50_ is marked with a red dotted line. The values are normalized mean values from three independent experiments. Ethanol 1% is used as control.

The antibacterial activity of aurofusarin against *L. acidophilus* was further investigated using strains of four additional *Lactobacillus* species and two of the related genus *Bifidobacterium.* Aurofusarin inhibited growth of all strains with IC_50_ values of: *Lactobacillus acidophilus* 8 µM, *Lactobacillus reuteri* 64 µM, *Lactobacillus salivarius* 32 µM, *Lactobacillus sobrius* >128 µM, *Bifidobacterium breve* 64 µM, and *Bifidobacterium longum* 128 µM (Figure 1).

**Figure 2:**
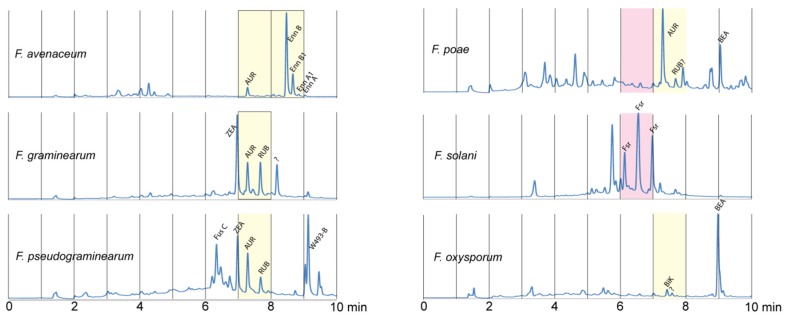
Isolated fractions with antibacterial effect. Secondary metabolites from each fungus were fractionized by preparative HPLC. Fractions in red showed antibacterial effect against *Staph. aureus.* Fractions in yellow showed antibacterial effect against *L. acidophilus*. *F. avenaceum* was grown on YMA, *F. graminearum* on PDA, *F. poae* and *F. pseudograminearum* on YES and *F. oxysporum* on rice. AUR: Aurofusarin. Enn: Enniatins. ZEA: Zearalenone. RUB: Rubrofusarin. BEA: Beauvericin. Fus C: Fusarin C. BIK: Bikaverin. ?: unknown.

The oCelloScope system was then examined for the potential as a tool for bio-guided identification of antibacterial secondary metabolites from *Fusarium*. In this test, 14 different *Fusarium* species were grown on four different media to enable the production of a wide selection of *Fusarium* secondary metabolites. The species were chosen to cover the phylogenetic diversity [24] and secondary metabolite potential [7] of the *Fusarium* genus. The resulting extracts were used in a screen against *L. acidophilus*, *E. coli*, *Staph. aureus* and *Salm. typhimurium*, in which extracts from six *Fusarium* species exhibited antibacterial activity. The active extracts were separated by HPLC into 10 fractions, which were subsequently screened individually. Two of the resulting fractions (from *F. poae* and *F. solani*) contained compounds with an effect against *Staph. aureus* (Figure 2).

The active fraction from *F. poae* did not contain a known compound detectable by the HPLC method, whereas the fraction from *F. solani* contained various forms of the pigment fusarubin. This is in accordance with previous studies where fusarubins have been shown to inhibit growth of *Staph. aureus* [25]. Five fractions from *F. avenaceum*, *F. graminearum*, *F. poae*, *F. pseudograminearum* and *F. oxysporum* exhibited effects against *L. acidophilus*. The active fractions against *L. acidophilus* from *F. oxysporum* contained the mycelium pigment bikaverin, whereas the active fractions from *F. avenaceum*, *F. graminearum*, *F. poae* and *F. pseudograminearum* contained the mycelium pigment aurofusarin and the pathway intermediate rubrofusarin. An additional fraction from *F. avenaceum*, which contained enniatin B, B_1_, A_1_ and A, exhibited an effect against *L. acidophilus*. The majority of the fractions did not inhibit bacterial growth, which is also in agreement with the initial set-up experiment. The inactive fractions contain several of the compounds which did not exhibit antibacterial activity, including zearalenone, fusarin C and trichothecenes (diacetoxyscirpenol, deoxynivalenol, nivalenol, neosolaniol, T-2 toxin). Together these results show that the system is useful as a bio-guided tool to discover compounds with antibacterial activities.

The producers of aurofusarin, mainly *F. graminearum*, *F. avenaceum* and *F. culmorum*, are primarily found in temperate climate zones, whereas bikaverin is produced by *F. verticillioides*, *F. fujikuroi* and *F. subglutinans* which are found in subtopic zones. All these *Fusarium* species are major pathogens of cereals used for human and animal feed. Aurofusarin, causing the characteristic coloring of several *Fusarium* species, is synthesized by PKS 12 and is a dimeric naphthoquinone. It has previously been shown to affect chicken egg quality and to possess antifungal effects [26,27]. The regulation of aurofusarin remains unknown but nitrogen sources seem to influence the expression in *Fusarium graminearum* [28]. High levels of both aurofusarin (up to 47 mg/kg) and bikaverin (up to 90 mg/kg) have been reported in cereals [29,30], and aurofusarin has also been found in apples (166 mg/kg) [31]. Here, we show that aurofusarin and bikaverin have an antibacterial effect on strains of *Lactobacillus* and *Bifidobacterium*. Bikaverin has previously shown an antibacterial effect against *E. coli* [32]. Aurofusarin and other naphthoquinones have previously been shown to penetrate the outer membrane of Gram-positive bacteria due to their lipophilic properties [33,34]. Baker et al. tested 22 naphthoquinones, and 15 exhibited antibiotic activities against the Gram-positive *Staph. aureus* [34]. During our screening, we did not observe any effect against Gram-negative bacteria, but we did observe activity in some fungal extract fractions against *Staph. aureus* probably due to naphthoquinones. The *Lactobacillus* and *Bifidobacterium* strains require anaerobic conditions and this might enhance the effect of aurofusarin on Gram-positive bacteria.

*Lactobacillus* and *Bifidobacterium* strains are regarded as having a positive influence on the gut system in humans and other animal species such as swine [35,36]. Inhibition of *Lactobacillus* and *Bifidobacterium* might cause a shift towards more harmful bacteria in the gut and thus result in diarrhea or other gut diseases. Diarrhea is a significant problem for pig breeders and is typically caused by bacteria such as *E. coli* [37]. In theory, a concentration of 50 mg/kg aurofusarin in cereals may result in a concentration of 40–80 µM in the gut system, more than five times the concentration shown to affect *Lactobacillus acidophilus* in this study. Further investigations of the antibacterial effect of aurofusarin in an animal model could be highly relevant.

## 3. Conclusions

In this study, we used 17 different secondary metabolites from *Fusarium* to develop a fast microscopic method for the assessment of antibacterial activity. The system could be used in a bio-guided approach to determine antibacterial fractions in several *Fusarium* extracts. In our study we observed a strong antibacterial effect of aurofusarin against *Lactobacilli*, which can have harmful consequences for the gut microbiota.

## 4. Materials and Methods

### 4.1. Secondary Metabolites

The following SMs were purchased: antibiotic Y (BioAustralis, Smithfield, Australia), aurofusarin (Adipogen, Epalinges, Switzerland), beauvericin (BioAustralis, Smithfield, Australia), enniatin complex A, A_1_, B, B_1_ (BioAustralis, Smithfield, Australia), cyclosporine (Sigma Aldrich, Brøndby, Denmark), diacetoxyscirpenol (Sigma Aldrich, Brøndby, Denmark), deoxynivalenol (Sigma Aldrich, Brøndby, Denmark), fumonisin B_1_ (BioAustralis, Smithfield, Australia), fusaric acid (Sigma Aldrich, Brøndby, Denmark), moniliformin (Sigma Aldrich, Brøndby, Denmark), nivalenol (Sigma Aldrich, Brøndby, Denmark), neosolaniol (Sigma Aldrich, Brøndby, Denmark), T-2 toxin (Sigma Aldrich, Brøndby, Denmark), zearalenone (Sigma Aldrich, Brøndby, Denmark). Fusarielin A [38], fusarielin H [39] and fusarin C [40] were prepared as described in the respective publications. All metabolites were dissolved in ethanol 96% to a concentration of 51.2 mM.

### 4.2. Bacterial Strains

Four bacterial species were selected based on differences in morphology and as references to previous studies. The Gram-negative facultative aerobe bacterium, *Escherichia coli* (ATCC 25922), was chosen as a standard for monitoring the antibacterial effect of fungal compounds. The *Salmonella enterica* serovar *typhimurium* 3389-1 (DT12) was chosen as representative of Gram-negative bacteria that cause problems in European agriculture, it was isolated from a clinical case of salmonellosis in pigs (D. L. Baggesen at the Technical University of Denmark, National Veterinary Institute). *Staphylococcus aureus* (ATCC 29213), a Gram-positive bacterium was chosen to provide a reference to previous studies. *Lactobacillus acidophilus* (DSMZ 20079) was chosen as a representative of a Gram-positive bacterium found in the guts of humans and monogastric animals. In experiments with aurofusarin, the following bacteria species were used: *Lactobacillus acidophilus* (20079), *Lactobacillus salivarius* (20555), *Lactobacillus sobrius* (16698), *Bifidobacterium longum* subsp. *longum* Reuter (20219), *Bifidobacterium breve* (20213) (All bacteria were obtained from DSMZ, Braunschweig, Germany).

### 4.3. Fungal Strains

Representative strains of fourteen *Fusarium* species were analyzed in this study, (*F. avenaceum* IBT 41708, *F. cerealis* IBT 40125, *F. culmorum* IBT 2925, *F. equiseti* IBT 8752, *F. graminearum* NRRL 31084, *F. langsethiae* IBT 9951, *F. oxysporum* IBT8890, *F. poae* IBT 9999, *F. proliferatum* IBT 40647, *F. pseudograminearum* NRRL 1544, *F. sambucinum* IBT 2524, *F. solani* FGSC9596, *F. sporotrichioides* IBT 9948, and *F. venenatum* IBT 1197) selected from the IBT collection at the Technical University of Denmark, the Agricultural Research Service culture collection (NRRL), at the National Center for Agricultural Utilization Research in Peoria, Illinois, IL, USA or Fungal Genetic Stock Center, Kansas City, MO, USA.

### 4.4. Culture Media

*E. coli* and *Salm. typhimurium* strains were grown aerobically at 37 °C in Luria-Bertani (LB) medium (Merck, Darmstadt, Germany). *Staph. aureus* was grown aerobically at 37 °C in brain heart infusion (BHI) broth (Merck, Darmstadt, Germany), whereas Lactobacillus acidophilus was grown in Mueller Hinton, MSH broth (Merck, Darmstadt, Germany) at 37 °C. The *Bifidobacterium* and *Lactobacillus* strains were grown in anaerobic tubes with MRS-medium (*Lactobacillus*) and Colon-medium (*Bifidobacterium*) [41]. All bacteria were grown in 15 mL tubes without shaking. Four different fungal media were used: Yeast Extract Sucrose (YES) medium, Potato Dextrose agar (PDA), Yeast Malt agar (YMA) [42] and Rice medium (30 g of white rice and 50 mL H_2_O, autoclaved for 15 min at 121 °C).

### 4.5. Secondary Metabolite Fractionation

All *Fusarium* species were grown on petri dishes (90 mm) for two weeks in the dark at 25 °C on four different media (YES, YMA, PDA, Rice). Forty plugs (4 mm) were used for extraction from each agar plate with 5 mL extraction solvent (ethyl acetate:dichloromethane:methanol (3:2:1, vol/vol) with 1% formic acid) in an ultra-sonic bath for 45 min. Rice medium (10 g) was extracted with 15 mL extraction solvent. The extracts were then evaporated to dryness and re-dissolved in 1.5 mL ethanol and used for initial antibacterial screen.

Extracts with antibacterial properties were fractionated on an Agilent 1260 semi-preparative HPLC system equipped with a 150 × 10 mm Gemini 5 µm C6-Phenyl 110 Å column (Phenomenex, Torrance, CA, USA) using a flow of 5.000 mL/min and a linear water-acetonitrile gradient, where both were buffered with 50 ppm trifluoroacetic acid. The gradient started at 10% ACN, which was increased to 100% in 10 min and held for 2 min. 100 µL of each extract was injected and the entire 12 min run was divided in 10 fractions. The fractions were evaporated to dryness and re-dissolved in ethanol.

### 4.6. Antibiotic Susceptibility Tests

An overnight culture (0.1 mL) of bacteria was transferred to 8 mL of medium and incubated for 2 h (37 °C) to reach the exponential phase. The OD_600_ was measured by optical density (UV-3100 PC spectrophotometer; VWR, Herlev, Denmark) and the bacteria were diluted to a concentration of 7.8 × 10^5^ bacteria·mL^−1^. Beads were added (2 × 10^4^ 6-μm·beads/ml, microsphere standard, B-7277; Invitrogen, Naerum, Denmark) in order to focus the microscope. The bacteria were loaded onto an F-base microtiter plate (100 μL/well) (TPP; Sigma-Aldrich, Brondby, Denmark). All pure compounds and fractions of fungal SMs were added as 1 µL in ethanol to each well resulting in a final concentration of 1% ethanol in each sample. Antibiotic susceptibility tests (ASTs) were performed using the oCelloScope detection system (Phillips Bio Cell A/S, Allerød, Denmark). This is a digital time-lapse microscopy scanning through a fluid sample, which generates series of images [43]. Each well was scanned repeatedly every 10 min and 10 pictures are obtained per well. Time-lapse experiments, digital analysis, and image processing were conducted by use of the SESA algorithm as previous described [6,44]. The oCelloScope was placed within an Innova 44 incubator (New Brunswick Scientific) in order to keep the temperature constant at 37 °C. All experiments were done in triplicates. In the experiments with *Lactobacillus* and *Bifidobacterium*, we used an anaerobe cabinet to set up the experiment and sealed each well in the plates with oil.

### 4.7. Digital Analysis

Time-lapse experiments, digital analysis, and image processing were conducted by a custom automation script in MATLAB version 8.0.0.783 (R2012b; The MathWorks, Inc., Natick, MA, USA). The BCA (Background Corrected Absorption) algorithm was developed as an equivalent to OD with increased sensitivity. The algorithm determines the growth kinetics using a background correction mask subtracted from the first scan and partial image histogram summation.

### 4.8. Statistical Analysis

All data are expressed as mean values. IC_50_ values are defined as minimum of tested concentrations to inhibit growth by 50%. GraphPad Prism version 6.00 for Windows (GraphPad Software, San Diego, CA, USA) was used for statistical analysis.

## Figures and Tables

**Table 1:** IC_50_ values for the five secondary metabolites with antibacterial effect. Bacteria were incubated with of secondary metabolites (2–256 µM) for 6 h (-: no effect).

	*L. acidophilus*	*E. coli*	*Staph. aureus*	*Salm*. *typhimurium*
Antibiotic Y	-	-	32 μM	-
Beauvericin	32 μM	-	32 μM	-
Enniatin mix	32 μM	-	64 μM	128 μM
Fusaric acid	64 μM	64 μM	-	-
Aurofusarin	8 μM	-	-	-

## Supplementary Materials

The following are available online at www.mdpi.com/2072-6651/8/12/355/s1, Figure S1: The inhibition effects of 16 different secondary metabolites on *L. acidophilus*, Figure S2: The inhibition effects of 16 different secondary metabolites on *E. coli*, Figure S3: The inhibition effects of 16 different secondary metabolites on *Staph. aureus*, Figure S4: The inhibition effects of 16 different secondary metabolites on *S. tryphimurium*. Video S1: Growth curve and videos of *Staph. aureus* grown with or without antibiotic Y. *Staph. aureus* were incubated in a 96 wells microtiter plate and scanned by oCelloScope every 10 min for 12 h.
