# Supplementary material for: Fast Screening of Antibacterial Compounds from Fusaria
Source: Toxins (Basel). 2016 Nov 29;8(12):355. doi: 10.3390/toxins8120355 (PMC5198549; doi:10.3390/toxins8120355)
Supplement: Supplementary file 1 [file toxins-08-00355-s001.zip › toxins-162560 supplementary/toxins-162560 supplementary.docx]

Supplementary Materials: Fast Screening of Antibacterial Compounds from Fusaria

Teis Esben Sondergaard, Marlene Fredborg, Ann-Maria Oppenhagen Christensen,
Sofie K. Damsgaard, Nikoline F. Kramer, Henriette Giese and Jens Laurids Sørensen








**Figure S1.** The inhibition effects of 16 different secondary metabolites on *L. acidophilus.* Sixteen different secondary metabolites were tested in the concentrations between 2 and 128 µM. The test was performed with the oCelloScope real-time microscopy program in 96-well standard plates. The values are normalized mean values from three independent experiments. Ethanol 1% is used as control.








**Figure S2.** The inhibition effects of 16 different secondary metabolites on *E. coli.* Sixteen different secondary metabolites were tested in the concentrations between 2 and 128 µM. The test was performed with the oCelloScope real-time microscopy program in 96-well standard plates. The values are normalized mean values from three independent experiments. Ethanol 1% is used as control.





**Figure S3.** *Cont*.





**Figure S3.** The inhibition effects of 16 different secondary metabolites on *S. aureus.* Sixteen different secondary metabolites were tested in the concentrations between 2 and 128 µM. The test was performed with the oCelloScope real-time microscopy in 96-well standard plates. The values are normalized mean values from three independent experiments. Ethanol 1% is used as control.








**Figure S4.** The inhibition effects of 16 different secondary metabolites on *S. tryphimurium.* Sixteen different secondary metabolites were tested in the concentrations between 2 and 128 µM. The test was performed with the oCelloScope real-time microscopy program in 96-well standard plates. The values are normalized mean values from three independent experiments. Ethanol 1% is used as control.
